# Identifying Myeloid‐Derived Suppressor Cells and Lipocalin‐2 as Therapeutic Targets for Intervertebral Disc Degeneration

**DOI:** 10.1002/advs.202500505

**Published:** 2025-06-26

**Authors:** Changmeng Zhang, Haoyun Li, Hongfei Wang, Liangyu Shi, Ying Shing Chan, Yu Wang, Graham Ka Hon Shea

**Affiliations:** ^1^ Department of Orthopaedics and Traumatology School of Clinical Medicine Li Ka Shing Faculty of Medicine The University of Hong Kong Hong Kong Hong Kong; ^2^ Department of Pharmacology and Pharmacy Li Ka Shing Faculty of Medicine The University of Hong Kong Hong Kong Hong Kong; ^3^ School of Biomedical Sciences Li Ka Shing Faculty of Medicine The University of Hong Kong Hong Kong Hong Kong

**Keywords:** genome‐wide association study, intervertebral disc degeneration, Lipocalin‐2, myeloid‐derived suppressor cell, single‐cell RNA sequencing

## Abstract

Inflammation is a hallmark of intervertebral disc degeneration (IVDD) characterized by immune cell infiltration and cytokine secretion. Stage‐specific transcriptomic analyses of IVDD via single‐cell RNA sequencing (scRNA‐seq) have primarily focused on nucleus pulposus cell phenotypes but not immune subpopulations. In other disease contexts, integrating genome‐wide association studies (GWAS) with scRNA‐seq data has provided insights on pathomechanisms in relation to specific cellular subpopulations via single‐cell disease relevance scores (scDRS). However, such an approach remains to be applied to IVDD. Here, the stage‐ specific analysis of IVDD in relation to Pfirrmann grading revealed a key transition in immune cells from a preponderance of LCN2^high^ myeloid‐derived suppressor cells (MDSCs) during early degeneration to a surge of proinflammatory IL1B+ macrophages in advanced IVDD. scDRS implicated IL1B+ M1‐like macrophages as a GWAS risk‐enriched subpopulation associated with disease, while functional validation indicated an immunomodulatory effect of LCN2^high^ MDSCs via ANXA1‐mediated inflammation suppression. Accordingly, LCN2 knockout mice exhibit accelerated IVDD, whereas recombinant LCN2 promoted macrophage polarization in vitro to the reparative phenotype by enhancing ANXA1 / Arginase‐1 expression and countering LPS/IFN‐γ‐induced pro‐inflammatory phenotype. This work identifies LCN2^high^ MDSCs as an immunoprotective subpopulation in early IVDD and highlights a potential role of LCN2 as a novel therapeutic agent.

## Graphical abstract

Inflammatory dysregulation drives intervertebral disc degeneration via stage‐dependent immune cellular dynamics. Single‐cell transcriptomics and genetic risk mapping revealed a shift from LCN2^high^ myeloid‐derived suppressor cells maintaining disc repair in early stages to IL1B+ macrophage‐dominated pathology in advanced disease. Functional validation identified LCN2 as a critical immunomodulator, rescuing inflammatory imbalance via regulating ANXA1/Arginase‐1 expression, thus highlighting its therapeutic potential in degenerative disc disease.

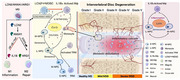

## 1 Introduction

Low back pain (LBP) is a global health concern of epidemic proportions affecting the adult population.^[^ ^1^ ^]^ Intervertebral disc degeneration (IVDD) represents both a pathological feature and underlying cause of LBP.^[^ ^2^ ^]^ The prevalence of radiological disc degeneration exceeds 90% in individuals aged 50 years and older.^[^ ^3^ ^]^ Resultant medical care expenses, lost wages, and reduced productivity amount to costs exceeding US $100 billion per year in the United States alone.^[^ ^4^ ^]^ With the ageing society being a growing issue especially in the developed world, the number of afflicted patients is expected to increase.

IVDD is characterized by complex cellular, molecular, and biomechanical changes within the disc. Existing evidence indicates that inflammation plays a pivotal role in the progression of disc degeneration.^[^^]^ Upon the onset of IVDD, nucleus pulposus (NP), annulus fibrosus, and immune cells secrete inflammatory cytokines, including tumor necrosis factor (TNF)‐α, interleukin (IL)‐1 α/β, IL‐6, and IL‐17. Elevated cytokine levels promote discal extracellular matrix degradation, recruitment and activation of inflammatory cells, as well as cell senescence.^[^ ^6^ ^]^ The hypoxic and inflammatory microenvironment within the degenerating disc promotes neovascularization, which facilitates further infiltration of immune populations from the systemic circulation.

Whilst numerous studies have described the presence of immune cells within degenerated human disc specimens,^[^ ^7^, ^8^ ^]^ comprehensive single‐cell transcriptomic analysis of these subpopulations from normal to diseased intervertebral discs (IVDs) is lacking. As inflammatory possesses play a pivotal role in IVDD pathophysiology,^[^ ^8^ ^]^ characterizing stage‐specific changes in immune subpopulations promises to increase our understanding of disease and to identify novel therapeutic avenues.

Here, we constructed a comprehensive human intervertebral disc atlas comprising of single‐cell RNA sequencing (scRNA‐seq) data spanning neonatal discs to healthy and severely degenerated adult specimens. We then utilized single‐cell disease relevance scores (scDRS) to associate scRNA‐seq results with IVDD‐associated genes implicated in genome‐wide association studies (GWAS).^[^ ^9^ ^]^ scDRS represents a means of attributing disease causality to specific cellular populations characterized at a single cell level. Through in silico gene perturbation modeling, an animal knockout model, and in vitro culture assays, we validated the function of candidate genes and proteins within disease‐associated cell subpopulations.^[^ ^10^ ^]^ Our results consolidated the role of ILB+ macrophages (Mφs) in driving the progression of disc degeneration and identified LCN2^high^ MDSCs and LCN2 as novel immunomodulatory treatment targets attenuating IVDD.

## 2 Results

### 2.1 Constructing a Single‐Cell Transcriptomic Atlas of Embryonic and Adult Human Intervertebral Discs

**Figure 1:**
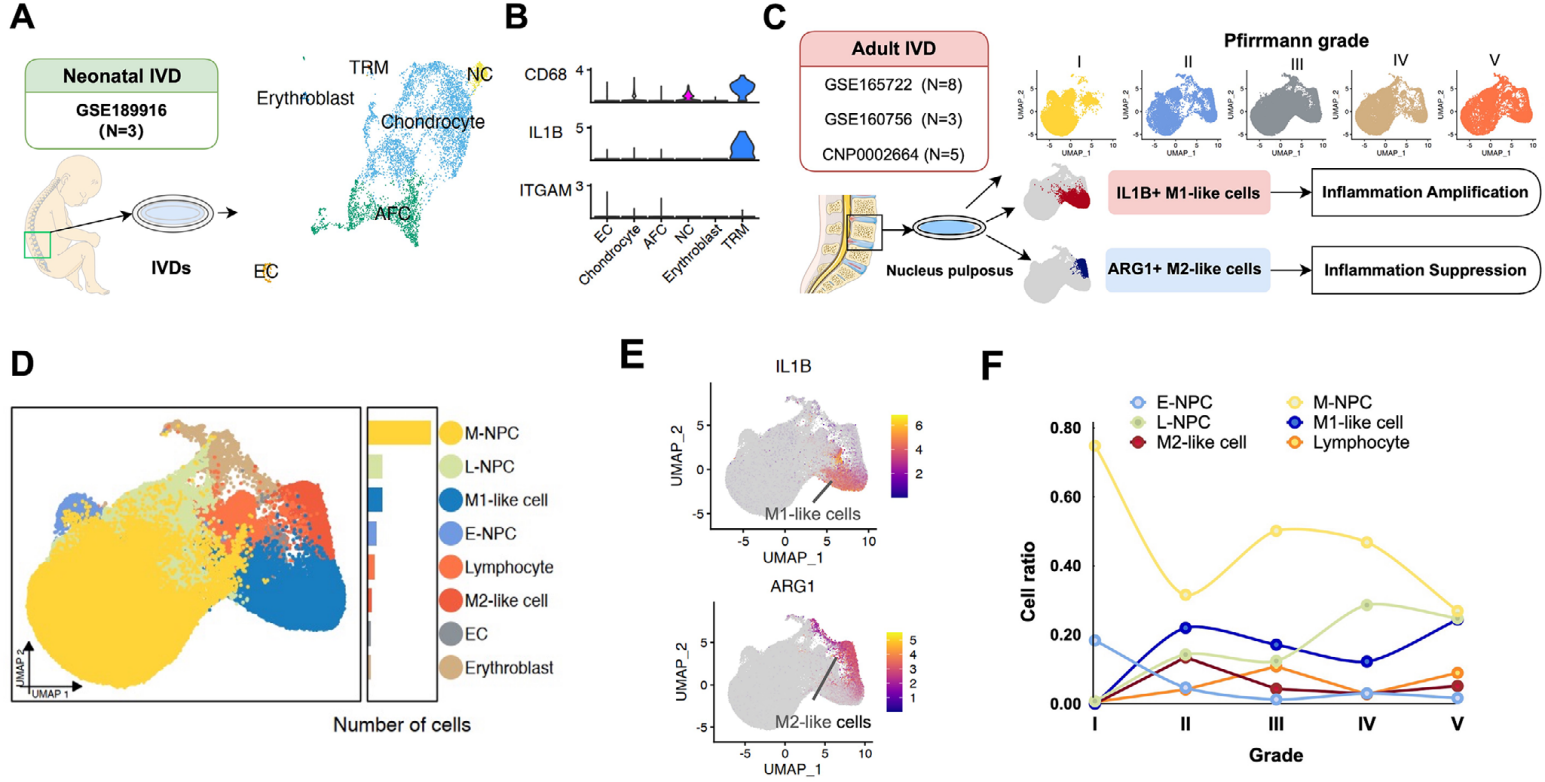
Constructing a single‐cell transcriptome atlas of the human intervertebral disc during health and disease. A) Integrated scRNA‐seq analysis of N = 3 neonatal human IVDs. The UMAP plot illustrated unbiased clustering of 6541 cells, resolving populations of notochordal cells (NCs), chondrocytes, annulus fibrosus cells (AFCs), endothelial cells (ECs), tissue‐resident macrophages (TRMs), and erythrocytes. B) Violin plot showing high expression of CD68 and IL1B and minimal expression of ITGAM in TRMs within the neonatal IVD. C) scRNA‐seq analysis workflow for N = 16 adult nucleus pulposus samples. Datasets were integrated and then categorized based on radiological Pfirrmann grading. Two immune cell predominant subpopulations could be identified across samples, namely IL1B+ M1‐like cells and ARG1+ M2‐like cells. D) UMAP plot of 100801 cells from 16 adult NP samples, displaying 8 identified cell clusters: main nucleus pulposus (NP) cells (M‐NPC), early NP cells (E‐NPC), late NP cells (late L‐NPC), M1‐like cells, M2‐like cells, and endothelials cells (EC). E) Gene expression levels of IL1B and ARG1 overlaid on UMAP plot, which was concentrated in M1‐like and M2‐like cell clusters respectively. F) Line chart depicting the change in ratio of cell types (1.0 = 100% of cells) across different Pfirrmann grades.

We first constructed a transcriptional atlas of human IVDs spanning from healthy neonatal (N = 3) to adult specimens (N = 16) at various stages of IVDD. Integration and unsupervised clustering upon neonatal IVD datasets identified a distinct subpopulation of KRT8+KRT18+ notochord cells (NCs) alongside the predominant chondrocyte‐like cell population (**Figure** **1**A; Figure S1A, Supporting Information). A small population of tissue‐resident Mφs (TRMs) could be identified which expressed CD68 and IL1B^[^ ^11^ ^]^ but were negative for myeloid marker ITGAM (Figure 1B). Functional enrichment analysis indicated the association of TRMs with immunoregulation, response to stimuli and the defense response, with IL1B identified as the hub gene (Figure S1B,C, Supporting Information). We then integrated the adult nucleus pulposus datasets which ranged from radiological Pfirrmann grade I to V (Figure 1C). The ACAN+SOX9+COL2A1+ NPC population could be classified into main nucleus pulposus cells (M‐NPC) which were present in normal and degenerated IVDs, early‐degeneration NPCs present in Pfirrmann grade I‐II IVDs (E‐NPC), and late‐degeneration NPCs present in Pfirrmann grade II‐V IVDs (L‐NPC) (Figure 1D; Figure S1D–F, Supporting Information). The hypoxic microenvironment of the intervertebral disc results in a non‐classical Mφ phenotype^[^ ^8^ ^]^ with cells designated as M1‐ and M2‐like subpopulations based on marker expression (Figure 1E; Figure S1F, Supporting Information). M1‐like cells were characterized by elevated IL1B expression, whereas M2‐like cells expressed arginase‐1 (ARG1) (Figure 1E).

The proportion of immune cells and NPCs were analyzed in accordance with Pfirrmann grading (Figure 1F). Pfirrmann grade I IVDs contained negligible immune cell numbers and were abundant in M‐NPCs. In Pfirrmann grade II IVDs, numbers of M1‐like and M2‐like cells increased, L‐NPCs indicative of NPC senescence increased (Figure S1G, Supporting Information), whilst E‐NPCs and M‐NPCs decreased. As IVDD progressed to grade III and beyond, the number of M2‐like cells decreased. Pfirrmann grade II IVDs therefore represented a transitory stage where both M1‐like and M2‐like cells featured, when a pro‐inflammatory microenvironment had yet to be established.

### 2.2 Role of Myeloid‐Derived IL1B+ Mφs in the Progression of Intervertebral Disc Degeneration

**Figure 2:**
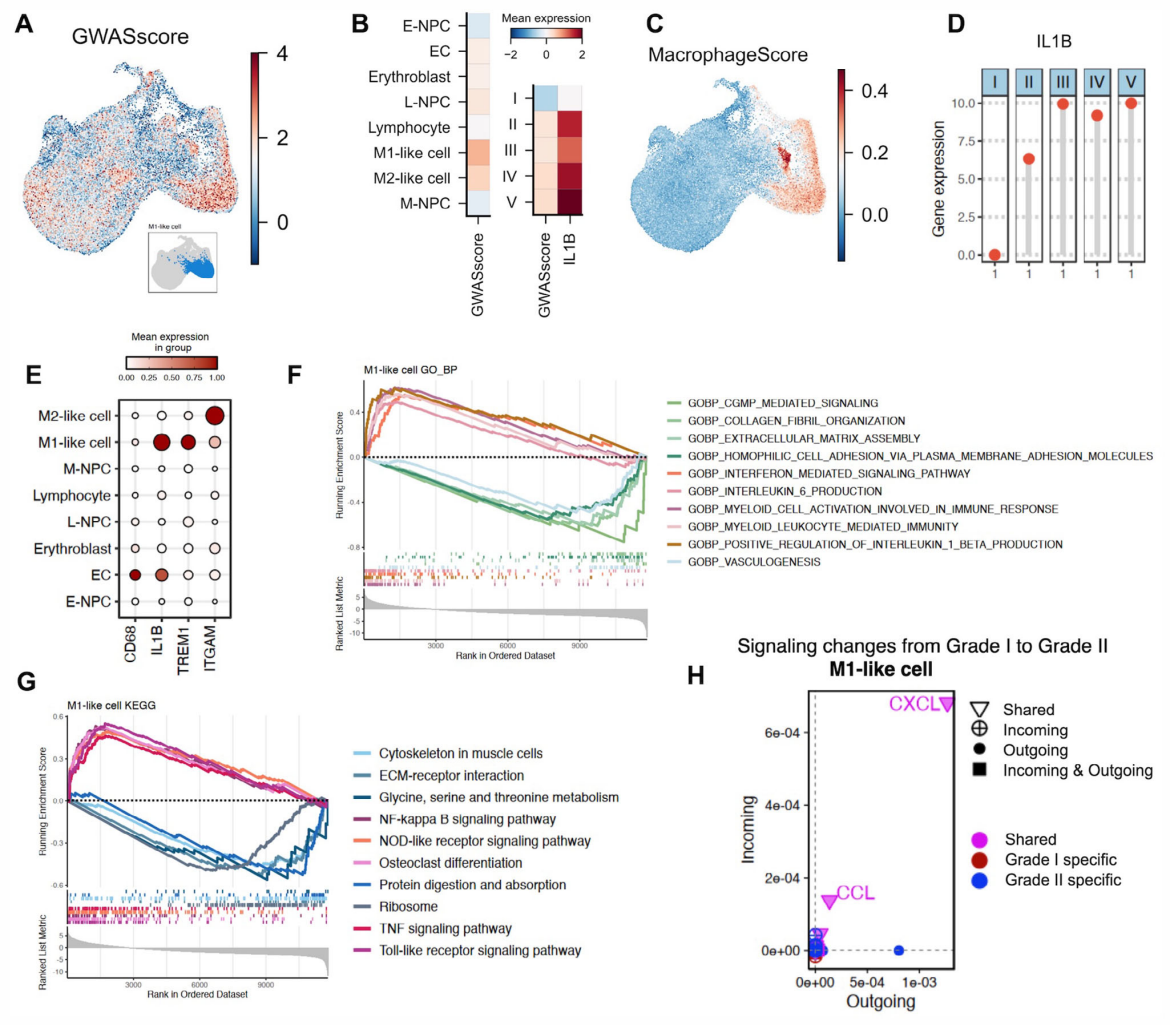
Role of myeloid‐derived IL1B+ macrophages in the progression of intervertebral disc degeneration. A) UMAP plot illustrating the enrichment of polygenic risk scores (GWAS scores) related to IVDD across different single cells in adult IVDs, calculated using scDRS. B) Heatmap showing the enrichment of GWAS scores across various cellular subpopulations and Pfirrmann grades. The trend in GWAS expression across Pfirrmann grades aligned with that of IL1B. C) Visualization of activated macrophage gene set enrichment scores (MacrophageScore) on UMAP. D) Lollipop chart showing IL1B gene expression at Pfirrmann grades I‐V. E) Dot plot showing elevated expression of IL1B and TREM1, with minimal expression of CD68 within the M1‐like subpopulation. GSEAplot facilitated enrichment analysis for M1‐like cells, showing F) GO biological process enrichment analysis and G) KEGG pathway analysis. H) 2D space plot indicating that CXCL and CCL signaling became the dominant outgoing/incoming signals within the M1‐like cell cluster at Pfirrmann grade II when compared to Pfirrmann grade I IVDs.

Single‐cell disease relevance scores (scDRS) allow for etiological association between GWAS and single cell data.^[^ ^9^ ^]^ We applied this approach to the scRNAseq datasets and a GWAS dataset comprising of 3039 individuals diagnosed with IVDD, and identified that polygenic disease risk was most enriched amongst M1‐like cells (**Figure** **2**A,B). As evidence for macrophage activation in IVDD, gene set “MacrophageScore” (GO:0 002281) projected onto the UMAP of the IVDD scRNA‐seq dataset demonstrated enrichment in expression amongst both M1‐like and M2‐like subpopulations (Figure 2C). Characterizing the expression of IL1B+ Mφs in adult IVDs suggested that cell numbers steadily increased with Pfirrmann grade (Figure 2D; Figure S2A, Supporting Information). Whilst adult IVDs did not express CD68, myeloid markers Triggering receptor1 (TREM1)^[^ ^12^ ^]^ and ITGAM (CD11B) were expressed in M1‐like and M2‐like cells respectively (Figure 2E). This provided evidence that a significant number of myeloid‐derived Mφs migrated within the degenerated nucleus pulposus.

We next conducted gene set enrichment analysis (GSEA) to understand the role of M1‐like cells during IVDD. Functional enrichment analysis revealed processes related to inflammation, including the recruitment and activation of myeloid cells and the production of pro‐inflammatory factors IL1B and IL6. Conversely, processes related to disc repair, such as collagen fibril organization, extracellular matrix assembly, cGMP‐mediated signaling, and angiogenesis were suppressed (Figure 2F). KEGG enrichment analysis similarly indicated the activation of inflammatory signaling pathways related to M1 activity, including the Toll‐like receptor pathway, NF‐kappa B (NF‐κB) pathway, and TNFα pathway (Figure 2G). Additionally, the CXCL and CCL signaling pathways (Figure 2H), crucial for the recruitment of neutrophils and monocytes,^[^ ^13^, ^14^ ^]^ abundantly featured. Analysis of cell‐cell interaction demonstrated M1‐like cells to be the predominant signaling sender/receiver within Pfirrman II and V IVDs, whilst they were the most active receiver in Pfirrmann grade III IVDs (Figure S2B, Supporting Information). Protein‐protein interaction (PPI) network analysis conducted upon M1‐like cells revealed IL1B as the central hub gene (Figure S2C, Supporting Information).

### 2.3 LCN2^high^ MDSCs Attenuate Inflammation During Intervertebral Disc Degeneration

**Figure 3:**
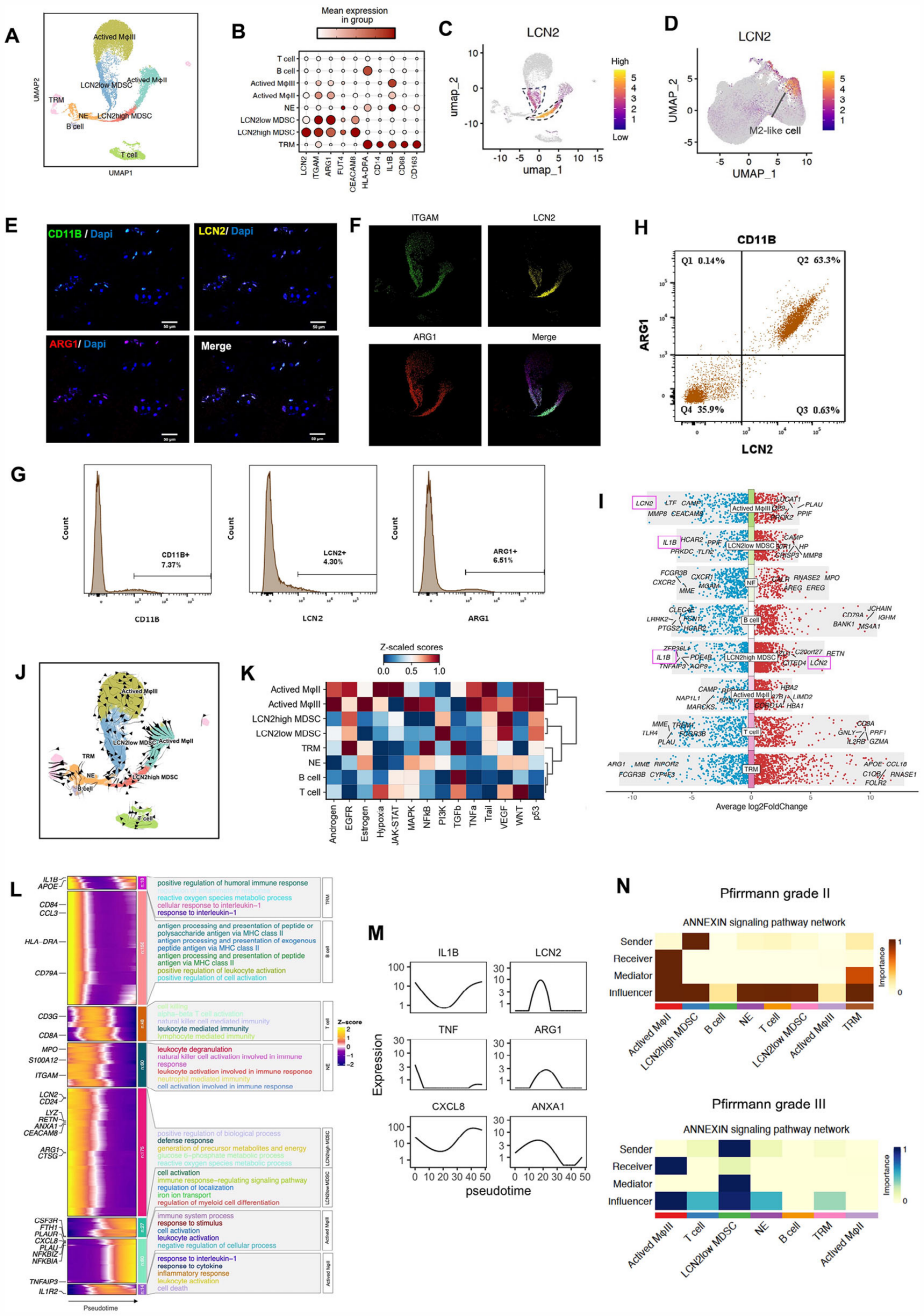
LCN2^high^ MDSCs mediate immune suppression during IVDD. A) UMAP visualization of 19136 re‐clustered immune cells from integrated adult nucleus pulposus (NP) datasets, resolving eight distinct populations: tissue‐resident macrophages (TRMs), Activated macrophages featuring at Pfirrmann grade II/III (Mφ II/III), LCN2^high^ and LCN2^low^ myeloid‐derived suppressor cells (MDSCs), and neutrophils (NE). B) Dot plot showing the expression levels of markers across different immune cells. MDSC clusters exhibited a polymorphonuclear MDSC (PMN‐MDSC) phenotype characterized by LCN2+CD11B(ITGAM)+ CD15(FUT4)+CD66b(CEACAM8)+ CD14‐HLA‐DR‐. C) UMAP illustrating LCN2 expression levels, with expression restricted to LCN2^high/low^ MDSC populations. D) LCN2 gene expression overlaid on UMAP containing all discal cells, showing enrichment in M2‐like macrophage clusters. E) Immunofluorescence staining of Pfirrmann grade IV‐V human intervertebral disc specimens demonstrating co‐expression of CD11B (green), LCN2 (yellow), and ARG1 (red) on MDSCs. F) UMAP of CD11B+, LCN2+, and ARG1+ cells, highlighting their co‐expression in MDSC populations. G) Flow cytometric quantification of CD11B+ (7.37%), LCN2+ (4.30%), and ARG1+ (6.51%) cells in human NP tissue specimens (N = 3). H) Dot plot showing that 63.3% of CD11B+ myeloid cells were triple‐positive for CD11B, LCN2, and ARG1. I) Volcano plot showing differentially expressed genes across different immune cell types. J) UMAP embedding of pseudotemporal cell trajectories inferred using the StaVia algorithm. (K) Heatmap depicting signaling pathway activities, with EGFR/VEGF pathways enriched in MDSCs and JAK‐STAT/NF‐κB/TNFα pathways in activated Mφs. L) Heatmap illustrating pseudotime‐aligned gene expression dynamics (left) and functional enrichment of differentially expressed genes (right) for each subpopulation. M) Pseudotime dynamics of LCN2, ARG1, ANXA1, IL1B, CXCL8, and TNF. N) Heatmap of ANXA1‐mediated signaling interactions between immune subpopulations (senders/receivers) in Pfirrmann grade II (upper panel) versus grade III (lower panel) discs.

Reclustering 19136 immune cells from Pfirrmann grade I – V IVDs with the exclusion of other cell types facilitated further characterization of grade‐specific inflammatory subpopulations and processes at greater resolution (**Figure** **3**A; Figure S3A, Supporting Information). In addition to M1‐like / M2‐like Mφs and lymphocytes, dimensionality reduction revealed activated Mφs subtypes II/III (according to their respective appearance at Pfirrman grade II and III IVDs, Figure S3D, Supporting Information), myeloid‐derived suppressor cells (MDSCs), and tissue resident Mφs (TRMs), the latter expressing CD14, IL1B, CD68 and CD163 (Figure 3B). Myeloid‐derived suppressor cells (MDSCs) are known to be essential in regulating adaptive and innate immunity in cancer and autoimmune disease^[^ ^15^ ^]^ but have not been characterized in IVDD. We observed that MDSCs differentiated into LCN2^high^ – and LCN2^low^‐MDSC subsets (Figure 3B,C). LCN2 is a secretory glycoprotein belonging to the lipocalin family important in the regulation of innate immunity^[^ ^16^ ^]^ that is expressed in the IVD^[^ ^17^ ^]^ and upregulated in response to axial loading.^[^ ^18^ ^]^ While LCN2 exhibited minimal expression in NP cells, it localized to within M2‐like cells across the integrated adult IVD datasets (Figure 3D). Following re‐clustering of immune cell subsets, these M2‐like cells were further subdivided into LCN2^high^‐ and LCN2^low^‐MDSC populations (Figure S3B, Supporting Information), indicating LCN2 expression to be markedly concentrated in MDSCs.

Three major subtypes of MDSCs may be identified in humans tissues: polymorphonuclear MDSCs (PMN‐MDSCs), monocytic MDSCs (M‐MDSCs), and immature MDSCs.^[^ ^19^ ^]^ LCN2^high^ MDSCs were analogous to PMN‐MDSCs (Figure 3B) in phenotype, expressing CD11B(ITGAM)+CD14‐CD15(FUT4)+ CD66B(CEACAM8)+HLA‐DR.^[^ ^19^ ^]^ LCN2^high^ MDSCs were abundant in Pfirrmann grade II discs relative to other stages (Figure S3B,C, Supporting Information), which suggested their capacity for an immunomodulatory role at a stage of early degeneration when the number of pro‐inflammatory M1‐like cells and IL1B expression remained low (Figures 1F and 2D). To provide supporting evidence that these immune populations and expression patterns persisted at the protein level, we performed immunofluorescence staining on human nucleus pulposus tissues, which revealed CD11B+LCN2+ARG1+ MDSCs (Figure 3E), a phenotype consistent with that of scRNA‐seq results (Figure 3F). Flow cytometry quantification demonstrated that CD11B+, LCN2+, and ARG1+ cells accounted for 7.37%, 4.30%, and 6.51% of total nucleus pulposus cells respectively (Figure 3G). Within the myeloid CD11B+ subpopulation, CD11B+LCN2+ARG1+ triple‐positive cells (MDSCs) constituted 63.3% of cells (Figure 3H).

Differential gene expression across immune cell subpopulations identified LCN2 as the most specifically expressed gene in LCN2^high^ MDSCs. IL1B expression was reduced in both LCN2^low^ and LCN2^high^ MDSCs whilst activated Mφ III present in Pfirrmann grade IVDs demonstrated reduced LCN2 expression (Figure 3I), which corroborated with our hypothesis that MDSCs were immunomodulatory at Pfirrmann grade II prior to the surge in IL1B expression. Pseudotime developmental trajectories indicated TRMs as the starting point, LCN2^high^ MDSCs occupying an intermediate state, and activated Mφ III as the differentiation endpoint to parallel transition of the immune landscape at the embryonic state to adult IVDDs from early to late‐stage degeneration (Figure S3E,F, Supporting Information). Single‐cell trajectory inference using StaVia similarly demonstrated progression of LCN2+ MDSCs toward inflammatory Mφ states during disease progression (Figure 3J), accompanied by sequential activation of NF‐κB, TNFα, and JAK‐STAT signaling cascades (Figure 3K).

Dynamic gene expression patterns with accordance to psuedotime indicated a role of IL1B in immune monitoring prior to disc degeneration and in inflammation amplification post‐degeneration. Conversely, MDSC‐associated markers (LCN2, ARG1, ANXA1) coordinated anti‐inflammatory responses and extracellular matrix remodeling (Figure 3L). Trajectory inference also indicated an elevated expression of inflammatory mediators (IL1B, CXCL8, PLAU) coupled with enhanced NF‐κB and TNFα pathway activation (Figure 3K,L). As the trend in anti‐inflammatory mediator expression (LCN2, ARG1, and ANXA1) was inversely correlated with pro‐inflammatory mediators (IL1B, CXCL8, TNF) along pseudotime (Figure 3M), this suggested that LCN2^high^ MDSCs coordinated immune suppression. Annexin A1 (ANXA1), present upon MDSCs, is a glucocorticoid regulatory protein that can suppress pro‐inflammatory signaling.^[^ ^20^ ^]^ GSEA/KEGG analyses further reinforced this regulatory pattern by demonstrating significant suppression of NF‐κB/TNFα pathways in LCN2^high^ MDSCs (Figure S3G,H, Supporting Information).

Cell‐cell communication analysis during the critical Pfirrmann grade II to III transition revealed that the shared signaling patterns among activated Mφs primarily were pro‐inflammatory, including IL1, IL6, TNF, and CXCL (Figure S3I,J, Supporting Information). In contrast, MDSCs predominantly signaled through the ANXA1‐FPR1 axis, a driver of reparative Mφ polarization,^[^ ^21^ ^]^ with activated Mφs serving as primary signaling targets (Figure 3N). ANXA1‐mediated signaling provided a putative mechanism for how MDSCs could counterbalance activated Mφs‐driven inflammation in IVDD.

### 2.4 Lipocalin‐2 Protects Against IVDD and is a Novel Therapeutic Target

**Figure 4:**
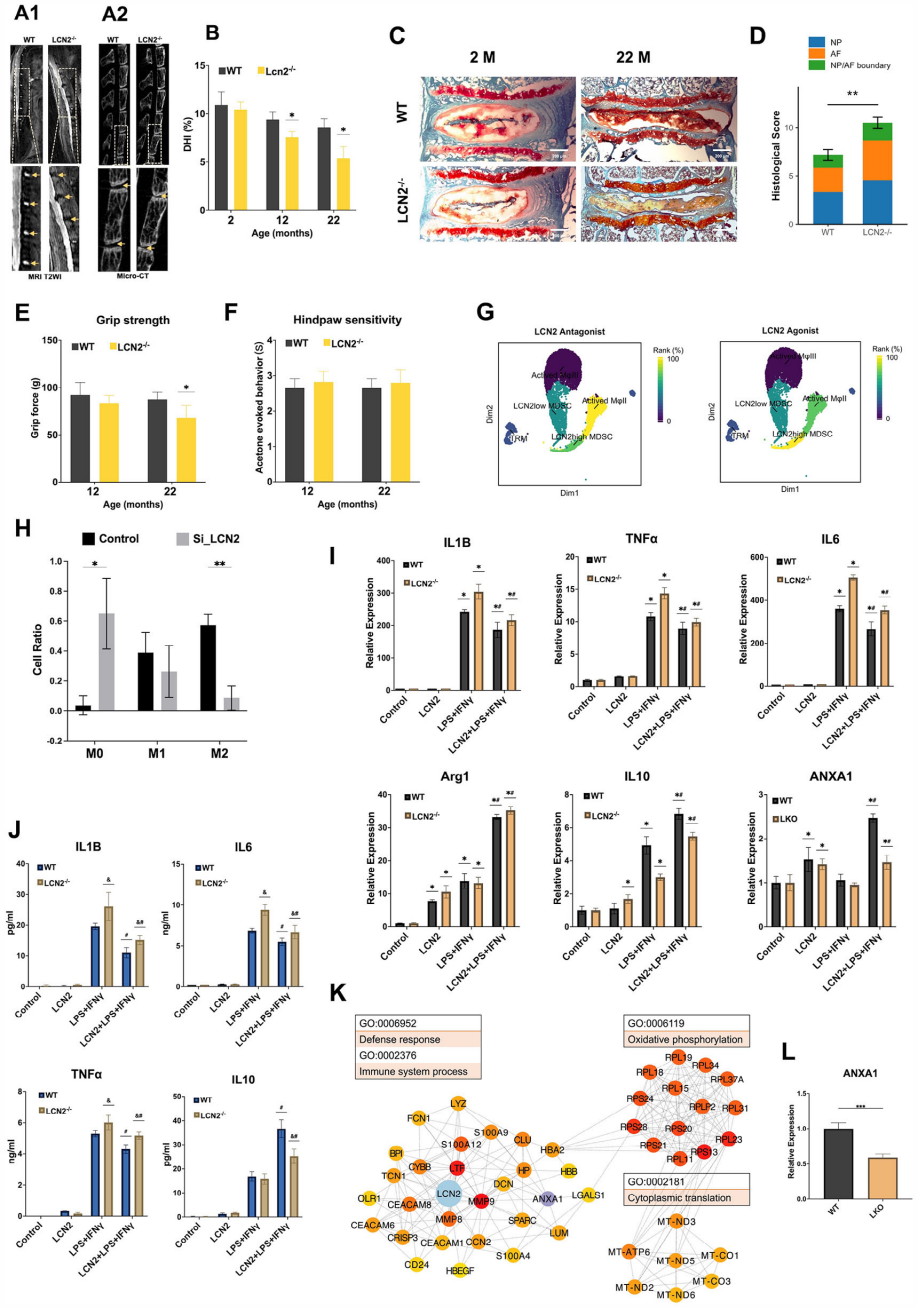
Targeting LCN2 as a novel therapeutic strategy for IVDD. MRI (A1) and micro‐CT (A2) images of aged (22‐month‐old) mice from wildtype (WT) and LCN2^−/‐^ mice, with magnification inset and yellow arrows indicating intervertebral disc spaces. B) Disc height index (DHI) calculated from micro‐CT at different ages in the two mouse genotypes, N = 6 per group, *P<0.05. C) Safranin‐O /Fast Green staining images of intervertebral discs from young (2‐month‐old) and aged (22‐month‐old) WT and LCN2^−/−^ mice (scale bars = 200 µm). D) Bar chart showing histological degeneration scores based on structural changes in the nucleus pulposus (NP), annulus fibrosis (AF), and NP/AF boundary. Aged LCN2^−/−^ mice exhibit significantly higher scores than WT, N  = 3 animals (6 discs) per group, *P < 0.01. E) Bar chart comparing grip force between groups across ages. N  = 6 per group, *P < 0.05. F) Acetone‐induced hindpaw sensitivity between groups showed no significant difference, N  = 6 per group. G) UMAP of human IVD immune cells with drug response inference: Activated Mφ II demonstrated highest sensitivity to LCN2 antagonists, while LCN2^high^ MDSCs responded most strongly to LCN2 agonists. H) Bar chart demonstrating reduced M2 macrophage polarization after LCN2 siRNA knockdown. N  = 3 per group. *P < 0.05, **P < 0.01. I) Quantitative RT‐PCR quantification of inflammatory markers in LCN2^−/‐^ versus WT bone marrow derived macrophages (BMDMs) under different culture conditions, N  = 4 per group; *P < 0.01 versus control from same group; **^#^** *P* < 0.01 versus LPS+IFNγ treatment within same group. J)  ELISA quantification of secreted proteins in BMDM culture supernatant. ^&^ *P* < 0.01 between groups under same treatment; **^#^** *P* < 0.01 versus LPS+IFNγ treatment within same group. K) PPI network displaying interactions among 73 perturbed genes in simulated LCN2 knockout human MDSCs, clustered into three functional modules. L) Bar chart showing reduced ANXA1 expression in LCN2^−/‐^ mouse MDSCs. N  = 4 per group, **P < 0.001.

We tested whether an absence of LCN2 would lead to accelerated IVDD in LCN2 knockout (LCN2^−/−^) mice (genotyping validation in Figure S4A, Supporting Information) by characterizing for early disc degeneration using magnetic resonance (MR) imaging, micro‐computed tomography (micro‐CT), and histological evaluation. Aged LCN2^−/‐^ mice (22 months old) exhibited increased desiccation and disc prolapse upon T2‐weighted MRI images in comparison to wildtype (WT) controls (**Figure** **4**A1, Supporting Information). Similarly, disc height index (DHI) upon micro‐CT evaluation showed a significant reduction in aged LCN2^−/−^ mice compared to the WT (Figure 4A2) by 12 months of age (Figure 4B). Histological evaluation corroborated with these radiological findings, with aged LCN2^−/−^ mice displaying narrowed intervertebral disc spaces, increased endplate (EP) ossification, and blurred boundaries between the annulus fibrosis (AF) and nucleus pulposus (Figure 4C). A standardized scoring system^[^ ^22^ ^]^ quantifying the aforementioned histopathological changes demonstrated significantly significant differences between WT and aged LCN2^−/−^ mice (Figure 4D). Immunohistochemistry against proteins associated with extracellular matrix dysregulation demonstrated diminished aggrecan (Acan) expression and elevated matrix metalloproteinase‐13 (MMP13) expression in aged LCN2^−/−^ mice IVDs (Figure S4B, Supporting Information). Furthermore, IL1B was absent in the discs of aged WT mice, whilst LCN2 expression could be seen over the ossified endplate. On the contrary, IL1B expression was present within the intervertebral discs of aged LCN2^−/−^ mice (Figure S4C, Supporting Information). Functional assessment for degeneration‐associated pain and behavioral manifestations was performed to correlate histopathological changes to clinical sequelae.^[^ ^23^ ^]^ Grip strength measurement, utilized as a behavioral index of axial pain tolerance, was significantly reduced in aged LCN2^−/−^ mice compared to WT controls (Figure 4E). Acetone sensitivity testing for radicular pain assessment revealed no significant differences both for adult / aged LCN2^−/−^ and WT mice (Figure 4F).

To evaluate the therapeutic potential of LCN2, we first employed scRank perturbation modeling on human IVD immune cell scRNA‐seq data.^[^ ^24^ ^]^ The cell type most responsive to LCN2 antagonism was the activated Mφ II population, while LCN2^high^ MDSCs exhibited the strongest response to LCN2 agonism (Figure 4G). Both subpopulations are predominantly present at Pfirrmann grade II (Figure S3D, Supporting Information), suggesting this stage as the optimal therapeutic window for LCN2‐targeted intervention. With reference to LCN2 expression patterns along pseudotime trajectory, the transition from LCN2^high^ MDSCs to activated Mφ II was accompanied by a marked downregulation of LCN2 (Figure 3J; Figure S4D, Supporting Information). Given the expression of LCN2 in M2‐like cells (Figure 3D), we investigated whether siRNA‐mediated LCN2 silencing in RAW264.7 cells affected macrophage phenotype, demonstrating that LCN2 silencing significantly reduced the ratio of cells exhibiting the M2 phenotype under RANKL‐stimulation (Figure 4H; Figure S4D,E, Supporting Information). Subsequently, we extracted bone marrow‐derived macrophages (BMDMs) from WT and LCN2^−/‐^ mice for induction toward the M1 phenotype. Quantitative RT‐PCR demonstrated enhanced pro‐inflammatory responses in LCN2^−/‐^ BMDMs, with elevated M1 markers (IL1B, IL6, TNF) and suppression of M2 markers (IL10, ARG1). Conversely, recombinant LCN2 provided to LPS/IFNγ‐stimulated BMDMs reversed this polarization bias, with upregulation of IL10/ARG1 expression and suppression of M1 markers (Figure 4I). ELISA of secretory proteins within culture medium confirmed an increase in reparative mediators and a decrease in inflammatory cytokines in response to LCN2 (Figure 4J). To infer the mechanism by which LCN2 exerts immunomodulatory effects in IVDD, we applied scTenifoldKnk ^[^ ^10^ ^]^ for the virtual knockout of LCN2 (LKO) in LCN2+ MDSCs. LKO simulations identified 73 significantly perturbed downstream genes, including ANXA1. Cluster analysis of these genes revealed three distinct modules, each corresponding to specific enrichment profiles: anti‐inflammatory, oxidative phosphorylation, and cytoplasmic translation (Figure 4K). Experimental validation of LKO simulation confirmed a reduction in ANXA1 expression in LCN2^−/−^ BMDMs (Figure 4L), while recombinant LCN2 treatment rescued expression of anti‐inflammatory ANXA1 in LPS/IFNγ‐stimulated BMDMs (Figure 4I).

## 3 Discussion

**Figure 5:**
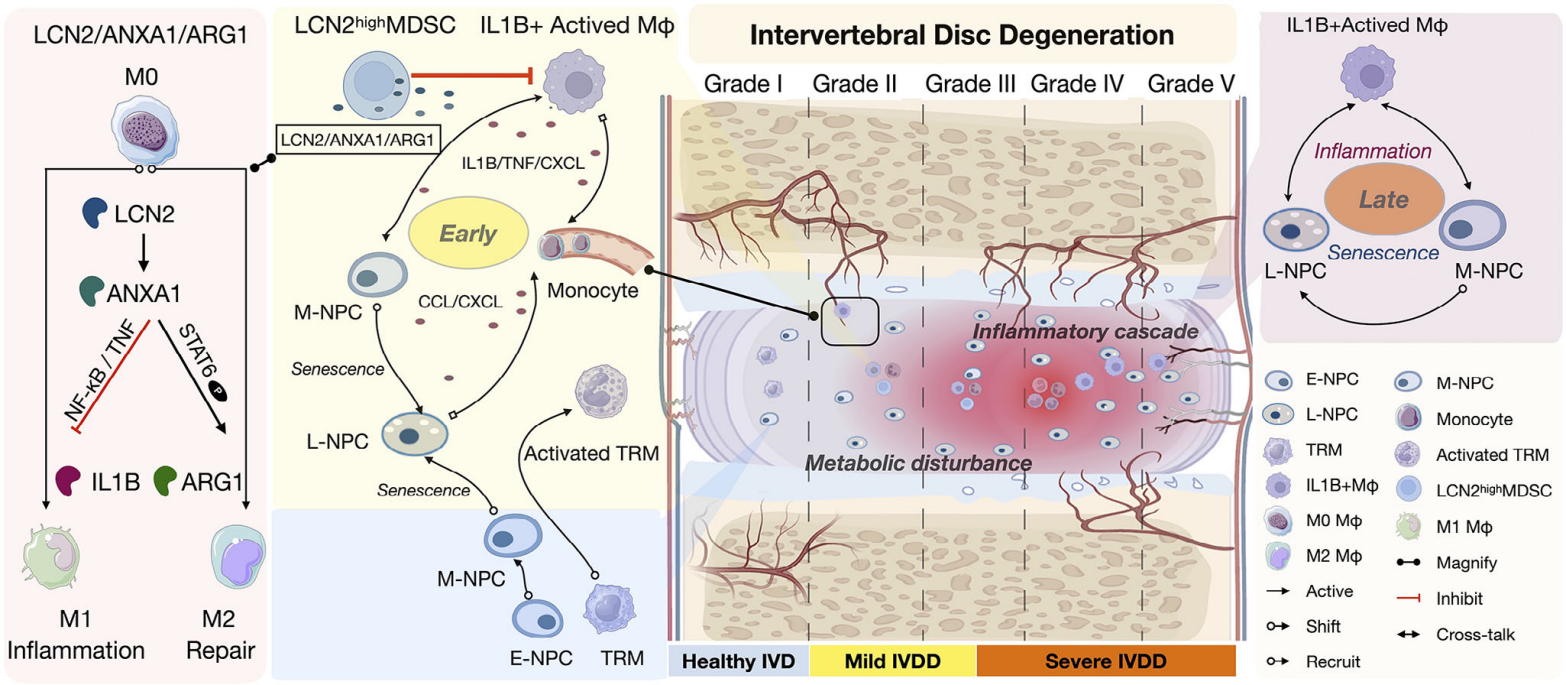
Schematic diagram illustrating the pathophysiology of IVDD with relation to inflammatory state and immune cellular phenotypes. LCN2^high^ MDSCs attenuate inflammation and promote tissue repair in early degeneration. Mechanistically, LCN2/ANXA1 exerts anti‐inflammatory effects by inhibiting NF‐κB/TNF signaling and promoting M2 polarization through induction of STAT6 phosphorylation, with subsequent ARG1 upregulation to facilitate tissue repair. More advanced degeneration is characterized by failed immunomodulation and IL1B+ macrophage‐driven disease progression, which has detrimental effects on NP cells leading to irreversible histopathological changes and a tissue remodeling response as opposed to repair.

In this study, we characterized cell populations and disease processes within the intervertebral disc in a stage‐specific manner. A summary of our findings is presented in **Figure** **5**. By integrating scRNA‐seq with GWAS using single‐cell disease relevance scores (scDRS),^[^ ^25^, ^26^ ^]^ we identified activated Mφ subpopulations enriched for IVDD‐associated genetic risk. Notably, LCN2^high^ MDSCs emerged as an immunomodulatory subpopulation in early‐stage IVDD and LCN2 was identified as a novel therapeutic target. These findings align with clinical and histological observations: healthy discs exhibit minimal resident immune cells whilst degenerated discs recruit myeloid immune populations, the latter being a double‐edged sword depending on whether LCN2^high^ MDSCs or IL1B+ proinflammatory Mφs predominate.

Non‐steroidal anti‐inflammatory drugs (NSAIDs) and corticosteroids have been administered both orally as well as intradiscally for the treatment of LBP. While these treatments provide therapeutic relief, systemic treatment in particular is associated with adverse outcomes^[^ ^27^, ^28^ ^]^ and it is unclear whether they can affect disease progression.^[^ ^5^ ^]^ It remains pertinent therefore, to identify therapeutic strategies in IVDD capable of attenuating disease and promoting repair. MDSCs have recently generated significant interest in the field of cancer immunology,^[^ ^29^ ^]^ and mediate immune suppression via amino acid depletion, nitric oxide production, and cytokine modulation.^[^ ^15^, ^30^ ^]^ Our work represents the first demonstration of LCN2^high^ mediated immunomodulation in IVDD as an endogenous protective mechanism and potential therapeutic target.^[^ ^31^ ^]^ Toward this objective, many preclinical studies have investigated the role of biomaterials,^[^ ^32^ ^]^ growth factors,^[^ ^33^ ^]^ biologics,^[^ ^34^ ^]^ and cells ^[^ ^35^ ^]^ on improving disc health, and our study add to this body of work. Critically, our results indicate the transition from Pfirrmann grade II to III discs as a prognostic checkpoint, beyond which ILB1+ M1 macrophage dominance and irreversible structural changes may render repair strategies futile.

LCN2 was initially recognized as an adipokine and pro‐inflammatory factor contributing to obesity‐related metabolic disorders.^[^ ^36^ ^]^ LCN2 has served as a biomarker for inflammation, ischemia, and infection.^[^ ^37^, ^38^ ^]^ The cellular origin of LCN2 affects its expression, potency, and function.^[^ ^16^ ^]^ With relation to macrophage function, LCN2 can also reduce the severity of graft‐versus‐host disease (GVHD) by decreasing MHCII expression and increasing IL‐10 production,  promoting M2 macrophage polarization by inducing STAT6 phosphorylation.^[^ ^39^, ^40^ ^]^ Our findings extend LCN2's protective functions to the intervertebral disc; previously with regards to the axial skeleton it has only been known to enhance hematopoiesis^[^ ^41^ ^]^ and delay bone marrow stromal cell (BMSC) senescence.^[^ ^42^ ^]^ From a mechanistic angle, our study demonstrated via in silico and in vitro assays that LCN2 synergizes with ANXA1 to suppress TNF/NF‐κB signaling during IVDD and promoting Mφ polarization toward the reparative M2 phenotype. Apart from being a key mediator of inflammation resolution and tissue repair,^[^ ^43^, ^44^ ^]^ ANXA1 is a gene characteristic of PMN‐MDSCs that allows them to be distinguished from classical neutrophils.^[^ ^45^ ^]^ Downstream of ANXA1,  PPARγ expression may be increased via STAT6, which can then inhibit NF‐kB‐mediated pro‐inflammatory responses.^[^ ^46^, ^47^ ^]^ We identified the reparative LCN2^high^ MDSCs present in early IVDD to be phenotypically analogous to PMN‐MDSCs, thus implicating LCN2‐ANXA1 crosstalk in MDSC‐mediated immunosuppression. While ANXA1's protective role in osteolysis has been established,^[^ ^20^ ^]^ this study is the first to define its functional association with LCN2^high^ MDSCs in the IVDD, thereby reinforcing LCN2‐ANXA1 axis as a mechanistically coherent therapeutic target for disc degeneration.  The dual regulatory role of LCN2‐ANXA1 in inflammation and metabolism from present and prior work underscores the novelty and biological relevance of LCN2 as a target for reversing early degeneration and preventing deterioration in IVDD.

Building upon our evidence that IVDs during early degeneration exhibit maximal responsiveness to LCN2 therapy, we propose a translatable strategy featuring the intradiscal injection of LCN2‐loaded reactive oxygen species (ROS)‐responsive hydrogel.^[^ ^48^ ^]^ A carrier suitable to the hostile disc milieu, characterized by inflammation, ischemia, and oxidative stress, is essential for sustained release of LCN2 such that progression to irreversible stages of degeneration (Pfirrmann grade IV/V) may be avoided. At present, our in vivo evidence is limited to the mouse model with its associated caveats. Whilst LCN2^−/−^ mice demonstrated accelerated age‐related degeneration of the IVD, we have yet to demonstrate rescue with intradiscal LCN2, and axial loads upon the spinal column differ in quadrupedal rodents from that of bipedal humans. Our animal model was also based on aging and degeneration which would differ to that of IVDD in response to injury and adjacent level disease after spinal fusion.^[^ ^49^ ^]^ Moreover, the absence of direct receptor‐binding studies, such as using LCN2‐neutralizing antibodies, impedes a more comprehensive understanding of protein function and downstream signaling in relation to IVDD. Furthermore, whilst the global LCN2 knockout model provides mechanistic insights, its broad expression necessitates future cell‐specific models, such as via macrophage‐targeted LCN2 knockouts. In future work, we aim to compare intradiscal LCN2 delivery in more robust animal models to attenuate disease as compared to existing pharmacological agents such as NSAIDs and corticosteroids, along with using LCN2‐neutralizing antibodies. With regards to bioinformatics analyses, ethnic heterogeneity in IVDD susceptibility is a confounder,^[^ ^50^ ^]^ and our assembled datasets prioritized Chinese cohorts, thereby affecting generalizability until future cross‐ethnic validation is performed.

## 4 Conclusion

By integrating a Pfirrmann grade‐specific scRNA‐seq atlas data with polygenic risk mapping, we characterized the immunological landscape in IVDD and identified LCN2^high^ MDSCs as a key modulator in early degeneration. Functional validation across computational, cellular, and preclinical models established the capacity of LCN2 to equilibrate pro‐inflammatory signaling via ANXA1‐mediated reparative pathways. Future work may be performed to consolidate findings upon different ethnicities and IVDD models, and toward establishing LCN2 as a novel therapeutic option not only to alleviate symptoms of LBP but to halt IVDD progression.

## 5 Experimental Section

### Data Sources

scRNA‐seq data was obtained from the Gene Expression Omnibus (GEO) database [GSE189916,^[^ ^51^ ^]^ GSE165722,^[^ ^52^ ^]^ GSE160756,^[^ ^53^ ^]^ GSE161125^[^ ^54^ ^]^] and the CellNetPortal (CNP) database [CNP0002664]^[^ ^55^ ^]^. These datasets included three neonatal disc samples^[^ ^51^ ^]^ and 16 adult nucleus pulposus samples from patients of Chinese descent^[^ ^52^, ^53^, ^55^ ^]^ ranging in age from 16 – 65 years and radiological Pfirrmann grade I to V. A bone marrow‐derived macrophage (BMDM) dataset from C57BL/6J mice was also included as a reference panel encompassing M0, M1, and M2 Mφ subtypes for comparison with in vitro culture assays.^[^ ^54^ ^]^ Table S1 (Supporting Information) contains detailed information about each sample.

The genome‐wide association study (GWAS) summary dataset for intervertebral disc degeneration (IVDD) was sourced from the China Kadoorie Biobank (CKB), a cohort comprising of 3039 individuals diagnosed with IVDD and 73642 control subjects of Chinese descent.^[^ ^56^ ^]^ All cases met the internationally recognized consensus criteria (ICD‐10) for IVDD diagnosis.

### Single‐Cell RNA Sequencing Analysis

scRNA‐seq analysis was conducted using the Seurat package (version 5).^[^ ^57^ ^]^ Quality control measures included the calculation of Unique Molecular Identifiers (UMIs) and the proportion of mitochondrial genes per cell, with dataset‐specific criteria for cell quality control listed in Table S1 (Supporting Information). Subsequently, single cell data were normalized using Seurat's LogNormalize method (scale factor = 10000) followed by log transformation. For downstream analysis, the top 2000 highly variable genes (HVGs) were selected. Labels were assigned to each sample based on the Pfirrmann grading.^[^ ^58^ ^]^ Neonatal (N = 3) and adult samples (N = 16) were integrated separately using the Seurat workflow for Anchor‐based Canonical Correlation Analysis (CCA) and involved CCA dimension reduction using the SelectIntegrationFeatures, FindIntegrationAnchors, and IntegrateData functions. Anchors were identified using 2000 variable features.

After integration, Shared Nearest Neighbors (SNN) graphs were constructed using the top 20 principal components (PCs) and visualized using Uniform Manifold Approximation and Projection (UMAP) embeddings. Clustering was performed using the FindNeighbors and FindClusters functions from the Seurat package. Differential gene expression (DEG) analysis was conducted using the FindMarkers function. Additionally, module scores for a defined set of genes in single cells were calculated using the AddModuleScore function in the Seurat package. scRNAtoolVis (https://github.com/junjunlab/scRNAtoolVis) and scCustomize R package (https://doi.org/10.5281/zenodo.5706430) were utilized to enhance the visualization of Seurat results.

For neonatal IVD cells, 6541 cells were classified into 14 subpopulations using unsupervised clustering methods. Specific markers were used to assign 6 cell types: notochordal cells (NCs), chondrocyte‐like cells, annulus fibrosus cells (AFCs), endothelial cells (ECs), tissue‐resident Mφs (TRMs), and erythrocytes. For adult NPCs, 100801 cells were clustered into 14 subpopulations. Marker genes were used to identify nucleus pulposus cells (NPC), lymphocytes, Mφ‐like cells, endothelial cells (ECs), and erythrocytes. For adult immune cells, 19136 cells were re‐clustered into 6 cell types: tissue‐resident Mφs (TRMs), myeloid‐derived suppressor cells (MDSCs), activated Mφs, B cells, T cells, and neutrophils (NE). Activated Mφs were further classified into activated MφII (predominantly in Pfirrmann grade II discs) and activated MφIII (predominantly in Pfirrmann grade III discs).

### Polygenic GWAS Signals on IVDD

The Single‐cell disease relevance score (scDRS) was utilized to link scRNA‐seq results to polygenic disease risk at single‐cell resolution.^[^ ^9^ ^]^ For gene‐level association analysis, the CKB IVDD GWAS summary data^[^ ^56^ ^]^ was preprocessed using MAGMA (v1.10) ^[^ ^59^ ^]^ with the following configurations: genome build hg19 (NCBI37.3.gene.loc), East Asian ancestry reference panel from the 1000 Genomes Project, and SNP‐to‐gene mapping within a 10 kb window surrounding gene bodies (5′ and 3′ extensions). This analysis produced gene‐level Z‐scores assessing disease associations, with full results available in Table S2 (Supporting Information).

In scDRS implementation (v1.0.1), the top 1000 MAGMA‐ranked genes were selected as putative disease genes following standard protocol.^[^ ^9^ ^]^ Each gene's contribution was weighted by its MAGMA Z‐score and adjusted for technical noise through mean‐variance modeling across scRNA‐seq data. Raw disease scores were normalized against 1000 Monte Carlo control gene sets that were matched for size, mean expression, and variance. Cell‐specific p‐values were derived from the empirical distribution of pooled normalized scores across all control sets and cells. The results were subsequently integrated into the scRNA‐seq object for visualization.

### GO Term and Pathway Enrichment Analysis

The g:Profiler web server (https://biit.cs.ut.ee/gprofiler/gost) was utilized to perform Gene Ontology (GO) term enrichment analysis. decoupleR was employed for pathway activity inference, with the reference being PROGENy, which encompasses curated pathways and their target genes.^[^ ^60^ ^]^

### Gene Set Enrichment Analysis (GSEA)

To comprehensively identify the biological activities of cell subpopulations, GSEA was performed on the sorted gene lists corresponding to respective cell subpopulations. This analysis was conducted using the R package clusterProfiler (version 4.0).^[^ ^61^ ^]^ The Gene Ontology Biological Process (GO_BP) enrichment analysis utilized gene sets from C5: ontology gene sets (downloaded from http://www.gsea‐msigdb.org/gsea/index.jsp), while KEGG signaling pathway annotations were obtained from https://rest.kegg.jp/list/pathway/hsa.

### Cell‐ Cell Communication Analysis

Intercellular communication analysis was conducted using the Cellchat R package (Version 2.1.0).^[^ ^62^ ^]^ Initially, CellChat was executed separately for each scRNA‐seq dataset representing different degeneration levels based on Pfirrmann grade. Subsequently, the CellChat objects were merged for downstream analyses. This approach allowed for insights to be gained into major signaling alterations occurring in cells during IVDD.

### Reconstruction of Cell Differentiation Trajectories

First Cytotrace (v0.3.3) was utilized to predict the relative differentiation status of individual cells and to identify the starting point of cellular differentiation.^[^ ^63^ ^]^ Next, Monocle3 was employed to facilitate the construction of single‐cell trajectory analyses and to characterize dynamic changes in gene expression during cellular differentiation.^[^ ^64^ ^]^ The inference of single‐cell developmental trajectories was performed using the StaVia package.^[^ ^65^ ^]^

### Construction of PPI Network

The STRING database (https://string‐db.org/) was utilized to create a protein‐protein interaction (PPI) network. The network was visualized using Cytoscape and the CytoNCA app (https://apps.cytoscape.org/apps/cytonca) to score protein interactions. To identify clusters of highly interconnected proteins, MCODE (https://apps.cytoscape.org/apps/mcode) was used which could represent functional modules and protein complexes.

### Drug Response Inference

scRank^[^ ^24^ ^]^was implemented to identify drug‐responsive cell populations within the IVDD scRNA‐seq dataset, enabling mechanistic characterization of LCN2‐targeted therapies at single‐cell resolution. This algorithm employs LCN2‐perturbed gene regulatory networks (GRNs) to computationally prioritize cell subtypes based on their predicted response profiles. Pharmacological interventions were specifically simulated through LCN2 antagonist and agonist perturbations. Spatial patterns of drug response probabilities were mapped onto UMAP embeddings.

### Virtual LCN2 Knockout in Human IVD

Virtual knockout of LCN2 was performed using scTenifoldKnk^[^ ^10^ ^]^ to evaluate its functional role in IVDD. The workflow comprised of reconstructing GRNs from the top 2000 highly variable genes within human IVDD scRNA‐seq data, virtually removing LCN2, performing manifold alignment between the perturbed and original GRNs to identify differentially regulated genes, and analyzing differentially regulated markers through PPI network construction and functional enrichment analysis.

### Cell Type Abundance Analysis

To assess Mφ phenotypic shift following LCN2 silencing, cell‐type abundance analysis was performed using CIBERSORT, a deconvolution algorithm that quantifies cell‐type composition from bulk transcriptomic data.^[^ ^66^ ^]^ Bulk RNA‐seq profiles of RANKL‐stimulated RAW264.7 Mφs treated with or without LCN2 small interfering RNA (siRNA) were obtained from GEO (GSE252232).^[^ ^67^ ^]^ Although RANKL‐activated M1‐type macrophages exhibit different properties compared to those induced by LPS+IFN‐γ, they provide a better representation of skeletal system degeneration in response to chronic inflammation.^[^ ^68^ ^]^ Data preprocessing, including normalization and log2 transformation, was conducted using the limma R package. A scRNA‐seq reference matrix encompassing M0, M1, and M2 Mφ phenotypes (Figure S4, Supporting Information) was utilized to resolve cellular fractions in bulk datasets via CIBERSORT, enabling quantitative assessment of M0/M1/M2 phenotypes under LCN2 silencing conditions.

### Human Tissue Sample Collection and Analysis

Human IVD tissues were obtained with informed consent from patients undergoing discectomy for lumbar degenerative disc disease (N = 6, age 57–73 years). All procedures were approved by the Institutional Review Board of the University of Hong Kong. Pfirrmann grade of the collected specimen was determined from pre‐operative MRIs (Table S3, Supporting Information). Surgical intervention amongst a symptomatic patient population dictated that all specimens exhibited advanced degeneration (Pfirrmann grade IV‐V).

For cellular phenotyping, NP tissues were minced and enzymatically digested using 0.25% pronase (Roche) followed by 200 U mL^−1^ collagenase II (Gibco). Isolated cells were filtered (70 µm strainer), stained with LIVE/DEAD Fixable Viability Dye (ThermoFisher), and labeled with surface marker CD11B. Intracellular targets (ARG1, LCN2) were stained after fixation (4% paraformaldehyde, PFA) and permeabilization (0.1% Triton X‐100). Details of antibodies utilized are provided in Table S4 (Supporting Information). Flow cytometry was performed on BD FACS Canto II (BD Biosciences) with IgG isotype controls for comparison. Data were analyzed using FlowJo software.

For tissue staining, harvested human disc tissues were fixed in 4% PFA, followed by dehydration and embedding in OCT tissue freezing medium (Leica). Frozen sections of 7 µm thickness were prepared using a cryostat (Thermo Fischer Scientific NX50). Immunofluorescence staining was performed as previously described.^[^ ^69^ ^]^ Briefly, sections were blocked in 3% bovine serum albumin (BSA, Sigma) for 1 h at room temperature, then incubated overnight at 4 °C with primary antibodies (Table S4, Supporting Information). After rinsing with PBS, the sections were incubated with fluorescent dye‐coupled secondary antibodies (Alexa Fluor) for 1 h. DAPI (Sigma) was used to stain the nuclei. Confocal microscopy (Zeiss LSM880) was employed to visualize the stained sections.

### Mice

Wild‐type (WT) and LCN2 knockout (LCN2^−/−^) mice were housed in a temperature‐controlled room set at 23 ± 1 °C with a 12‐h light‐dark cycle. Genomic DNA was extracted for PCR to confirm the absence of the LCN2 gene in knockout mice (Figure S4A, Supporting Information). Primers for genotyping are listed in Table S5 (Supporting Information). Animal experiments conducted in this study received approval from the institutional Committee on the Use of Live Animals in Teaching and Research (CULATR).

### MRI and Micro‐ CT

MRI scans (Bruker's PharmaScan) were performed in WT and LCN2^−/−^ mice at 22 months of age to evaluate for disc degeneration, whilst micro‐computed tomography (micro‐CT, Skyscan 1076) scans were conducted at 2, 12, and 22 months of age to measure intervertebral disc height. Image analysis and processing were performed using the 3D SUITE software. Intervertebral disc height was assessed by measuring disc height index (DHI).^[^ ^70^ ^]^

### Behavioral Assessment

Axial stretch hypersensitivity and cold sensitivity were evaluated as previous described.^[^ ^23^ ^]^ Grip strength (peak force in grams) was measured twice at 10 min intervals using a Grip Strength Meter (Sansbio, China) during tail‐induced stretching. Cold sensitivity was assessed by applying 25 µL acetone to the hind paw and recording nociceptive behavior duration within 1 min.

### Mice Tissue Sample Collection and Analysis

Lumbar vertebral discs from WT and LCN2^−/−^ mice at 2, 12, and 22 months of age were collected for histological analysis. Tissues first underwent decalcification in 0.5 m EDTA (pH 7.5, Sigma) after fixation in 4% PFA. Subsequent section preparation processing followed the protocols described for human specimens.

For Safranin‐O/Fast Green staining, sections were rehydrated via graded ethanol series and stained with 0.1% Fast Green (Sigma‐Aldrich) for 5 min, followed by 0.1% Safranin‐O (Merck) for 3 min. Sections were briefly differentiated in 1% acetic acid, dehydrated, and mounted in DePeX (BDH Laboratory). The stained sections were visualized using an Olympus BX43 microscope. A standardized IVD degeneration scoring system was applied to quantify histopathological changes across three compartments: nucleus pulposus (NP, 0–5), annulus fibrosus (AF, 0–6), and AF/NP boundary integrity (0‐2).^[^ ^22^ ^]^ Immunofluorescence staining was performed following the protocol detailed for human specimens.

### Bone marrow‐derived macrophage (BMDM) Isolation

BMDMs were isolated from 6–8 week old WT and LCN2^−/−^ mice. Femoral and tibial bone marrow was flushed out with cold PBS. After red blood cell lysis (ACK buffer, 5 min), cells were plated in BMDM medium (RPMI‐1640 medium supplemented with 10% FBS, 1% penicillin/streptomycin, 2 mm L‐glutamine) for 4 h at 37 °C and 5% CO2. Non‐adherent cells were transferred to 6‐well plates and differentiated with 20 ng ml^−1^ recombinant M‐CSF (Sino Biological) for 7 days prior to functional assays.

### Macrophage Polarization Assay

Primary BMDMs from mice were polarized on day 7 of differentiation. Lipopolysaccharide (LPS) and interferon‐gamma (IFN‐γ) were provided in medium for M1 polarization. Four experimental groups were established: 1) Control group: BMDM medium; 2) LCN2 group: BMDM medium + 500 ng mL^−1^ LCN2; 3) LPS+ IFN‐γ group: BMDM medium + 100 ng mL^−1^ LPS (InvivoGen) + 20 ng mL^−1^ IFN‐γ (Sigma); 4) LCN2+LPS+ IFN‐γ group: BMDM medium + 100 ng mL^−1^ LPS + 20 ng mL^−1^ IFN‐γ + 500 ng mL^−1^ LCN2. For groups receiving LCN2 (groups 2 and 4), cells were pre‐treated with LCN2 for 24 h prior to M1 polarization, and LCN2 supplementation was maintained throughout the subsequent 24‐h polarization period.

Following polarization, cells were harvested for real‐time quantitative PCR. For cytokine quantification, cell culture supernatants were collected (centrifuged at 300 × g for 10 min to remove debris) and analyzed for IL1B, IL6, TNFα, and IL10 secretion using commercial ELISA kits (Invitrogen) according to the manufacturer's protocols. Absorbance at 450 nm was measured using a BioTek plate reader (Winooski, VT, USA).

### Real‐Time Quantitative PCR Assay

Real‐time quantitative PCR (RT‐PCR) was performed to analyze gene expression in polarized Mφs from the treatment groups described above. Total RNA was extracted and purified using the RNeasy kit (Qiagen), and cDNA synthesis was carried out using PrimeScript reverse transcriptase (Takara) and Oligo‐(dt) primers following established protocols.^[^ ^69^ ^]^ For the RT‐PCR assay, SYBR Green Supermix (Bio‐Rad) was used on a Bio‐Rad CFX96 Touch R‐T PCR Detection System. Primers used in this study are listed in Table S6 (Supporting Information). To normalize the data, GAPDH mRNA was used as an internal control. The formula ΔCt = 2^ [Ct (target gene) – Ct (GAPDH)] was applied to calculate the relative expression levels of target genes.

### Statistical Analysis

Statistical analyses were performed using GraphPad Prism (v9.0, GraphPad Software). Normality was assessed before significance testing. Normally distributed data were analyzed using parametric tests, including unpaired two‐tailed Student's t‐test (two‐group comparisons) and one‐way ANOVA with Tukey's post hoc test (multi‐group comparisons). Non‐normally distributed data were evaluated with Mann‐Whitney U (two groups) or Kruskal‐Wallis tests (multi‐groups). Significance was defined as *p* < 0.05. All animal and cellular experiments included three biological replicates.

### Ethics Approval Statement

Animal experiments conducted in this study received approval from the institutional Committee on the Use of Live Animals in Teaching and Research (CULATR).

## Conflict of Interest

The authors declare no conflict of interest.

## Author Contributions

C.M.Z. and H.Y.L. contributed equally to this work. C.M.Z., H.Y.L., Y.S.C., Y.W., and G.K.H.S. conceived and designed the study. C.M.Z. and H.Y.L. performed the experiments. C.M.Z. and H.F.W. performed bioinformatics analysis. C.M.Z., H.Y.L., and L.Y.S. managed data collection and analysis. Y.S.C. and G.K.H.S. provided funding. C.M.Z. and G.K.H.S. wrote the paper. All authors reviewed and approved the final manuscript.

## Supporting information



Supporting Information

Supplemental Table 1

Supplemental Table 2

Supplemental Table 3

Supplemental Table 4

Supplemental Table 5

Supplemental Table 6

## Data Availability

The data that support the findings of this study are available from the corresponding author upon reasonable request.
