# Supplementary material for: Identifying Myeloid‐Derived Suppressor Cells and Lipocalin‐2 as Therapeutic Targets for Intervertebral Disc Degeneration
Source: Adv Sci (Weinh). 2025 Jun 26;12(34):e00505. doi: 10.1002/advs.202500505 (PMC12442675; doi:10.1002/advs.202500505)
Supplement: Supplementary file 1 — Supporting Information [file ADVS-12-e00505-s006.pdf]

ADVANCED SCIENCE
Open Access

Supporting Information

for *Adv. Sci.*, DOI 10.1002/advs.202500505

Identifying Myeloid-Derived Suppressor Cells and Lipocalin-2 as Therapeutic Targets for Intervertebral Disc Degeneration

*Changmeng Zhang, Haoyun Li, Hongfei Wang, Liangyu Shi, Ying Shing Chan, Yu Wang and Graham Ka Hon Shea**

Supporting Information

Identifying Myeloid-Derived Suppressor Cells and Lipocalin-2 as Therapeutic Targets for Intervertebral Disc Degeneration

*Changmeng Zhang, Haoyun Li, Hongfei Wang, Liangyu Shi, Yingshing Chan, Yu Wang, Graham Ka Hon Shea**

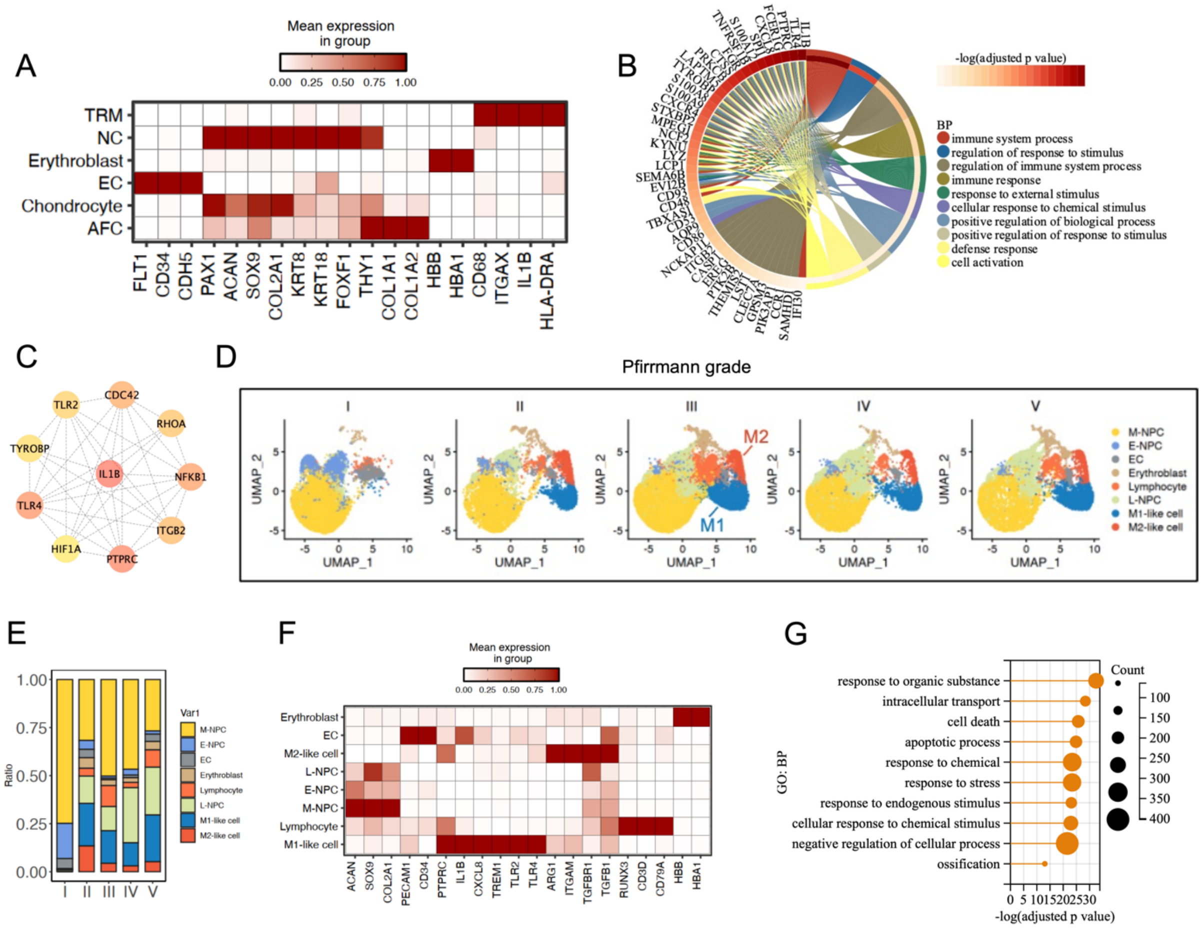

Figure S1. Constructing a single-cell transcriptome atlas of human intervertebral disc.

(**A**) Heatmap of integrated neonatal cell subpopulations (N=3) and specific marker expression. (**B**) Chord chart illustrated functional enrichment analysis of TRMs. **(C)** PPI analysis exhibiting the network relationships of the top 10 genes in TRMs, with IL1B identified as the core regulatory protein. **(D)** UMAP of integrated adult IVD samples divided by individual Pfirrmann grade. **(E)** Stacked bar charts depicting the ratio of each cell subpopulation within the adult IVD across Pfirrmann grades I-V. **(F)** The heatmap presenting comparative markers expression in different adult IVD cell subpopulations. **(G)** Lollipop chart demonstrated functional enrichment analysis of L-NPC cells.

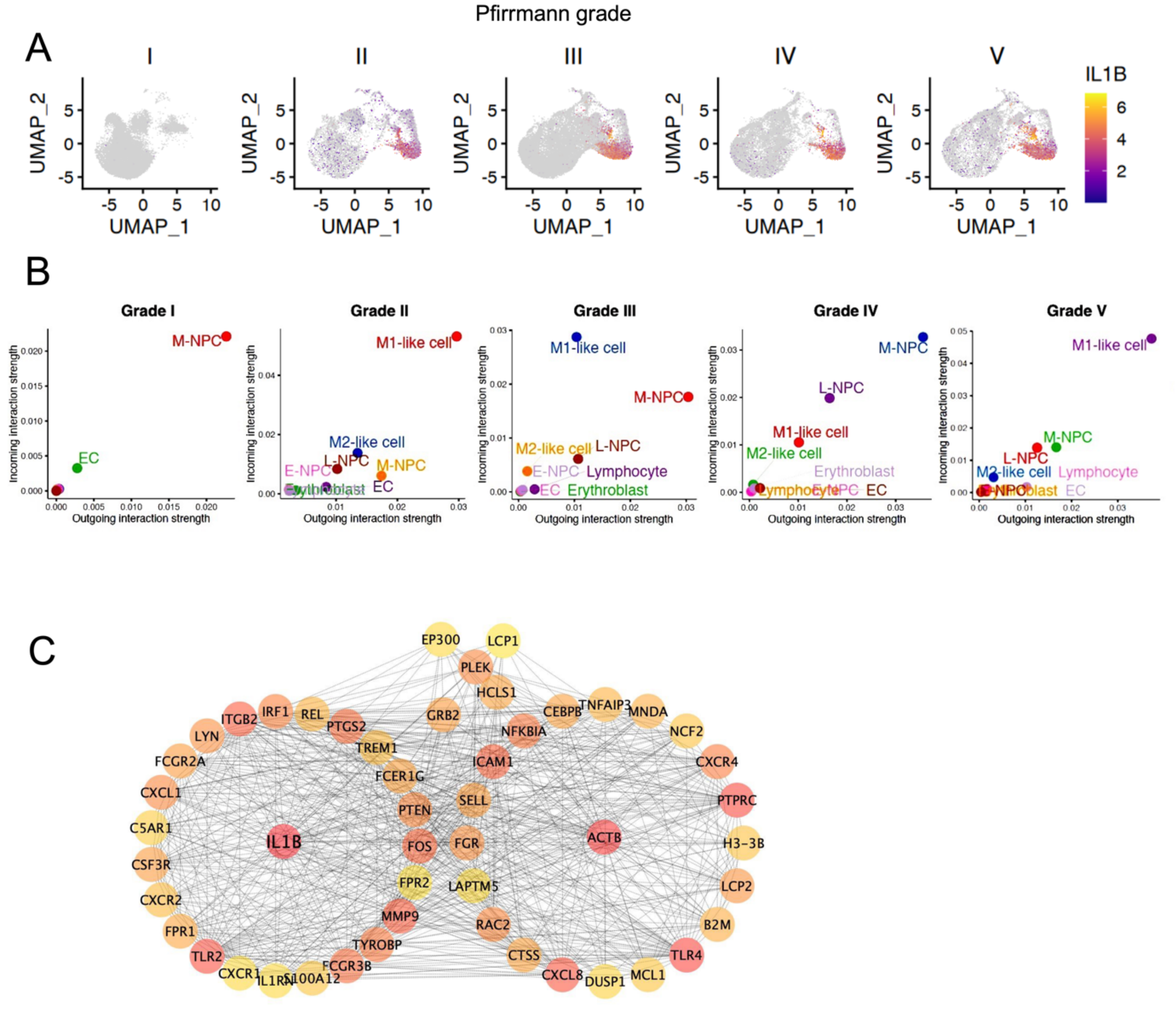

Figure S2. Role of myeloid-derived IL1B+ macrophages in the progression of intervertebral disc degeneration

(A) Split UMAP across Pfirrmann grades illustrating the distribution and abundance of IL1B+ cells. **(B)** Two-dimensional space plot showeing cell populations with significant changes in sending or receiving signals across Pfirrmann grades I-V. (**C)** PPI analysis displayed the network relationships of the top 50 genes in M1-like cells, with IL1B and ACTB identified as core regulatory proteins.

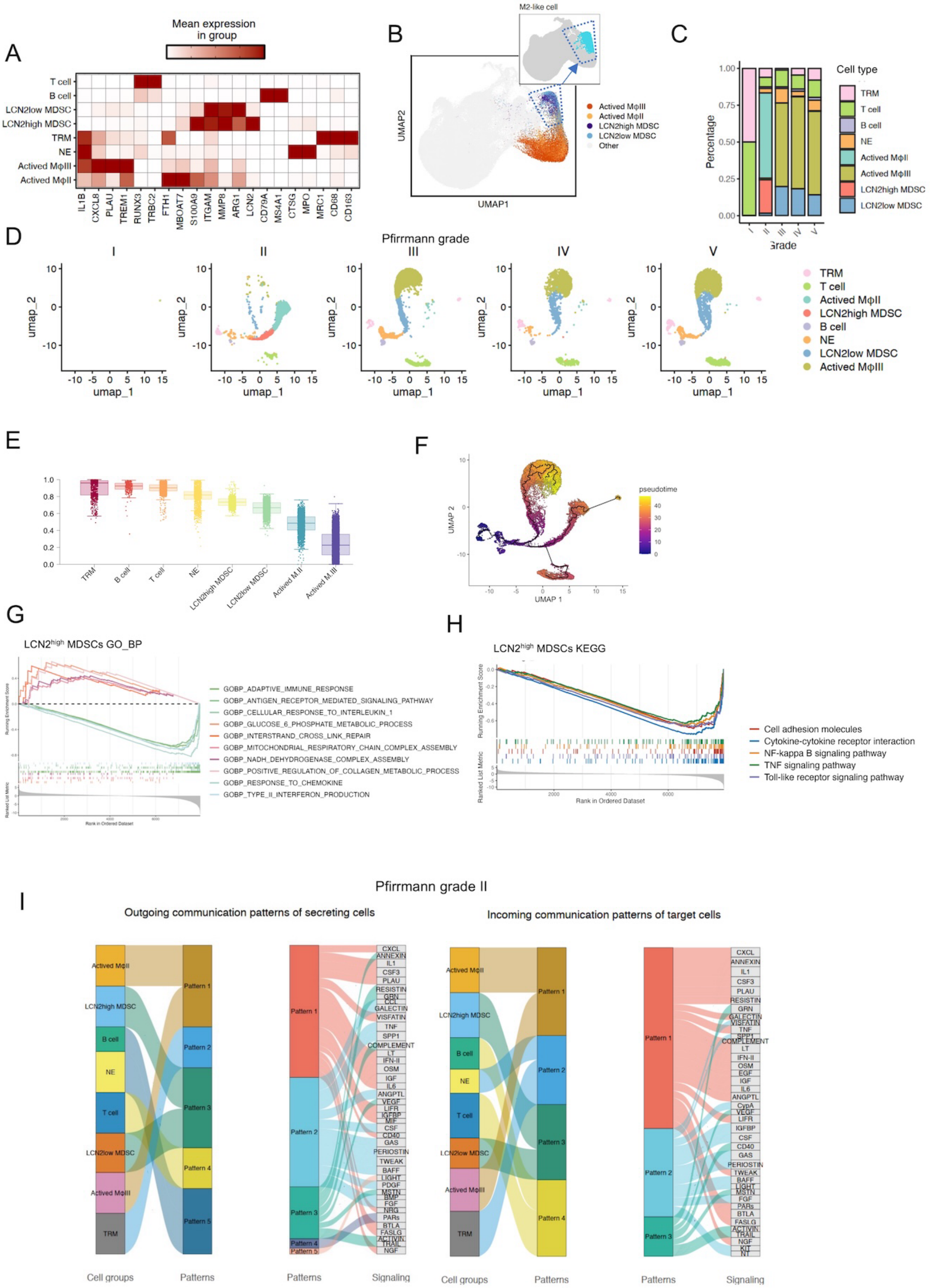
A
Mean expression in group
T cell
B cell
LCN2low MDSC
LCN2high MDSC
TRM
NE
Actived MφIII
Actived MφII
IL1B
CXCL8
PLAU
TREM1
RUNX3
TRBC2
FTH1
MBOAT7
S100A9
ITGAM
MMP8
ARG1
LCN2
CD79A
MS4A1
CTSG
MPO
MRC1
CD68
CD163
B
M2-like cell
UMAP2
UMAP1
Actived MφIII
Actived MφII
LCN2high MDSC
LCN2low MDSC
Other
C
Cell type
TRM
T cell
B cell
NE
Actived MφII
Actived MφIII
LCN2high MDSC
LCN2low MDSC
Percentage
Grade
D
Pfirrmann grade
I
II
III
IV
V
umap_2
umap_1
TRM
T cell
Actived MφII
LCN2high MDSC
B cell
NE
LCN2low MDSC
Actived MφIII
E
TRM
B cell
T cell
NE
LCN2high MDSC
LCN2low MDSC
F
pseudotime
UMAP 2
UMAP 1
G
LCN2high MDSCs GO_BP
Running Enrichment Score
Ranked List Metric
Rank in Ordered Dataset
GOBP_ADAPTIVE_IMMUNE_RESPONSE
GOBP_ANTIGEN_RECEPTOR_MEDIATED_SIGNALING_PATHWAY
GOBP_CELLULAR_RESPONSE_TO_INTERLEUKIN_1
GOBP_GLUCOSE_6_PHOSPHATE_METABOLIC_PROCESS
GOBP_INTERSTRAND_CROSS_LINK_REPAIR
GOBP_MITOCHONDRIAL_RESPIRATORY_CHAIN_COMPLEX_ASSEMBLY
GOBP_NADH_DEHYDROGENASE_COMPLEX_ASSEMBLY
GOBP_POSITIVE_REGULATION_OF_COLLAGEN_METABOLIC_PROCESS
GOBP_RESPONSE_TO_CHEMOKINE
GOBP_TYPE_II_INTERFERON_PRODUCTION
H
LCN2high MDSCs KEGG
Running Enrichment Score
Ranked List Metric
Rank in Ordered Dataset
Cell adhesion molecules
Cytokine-cytokine receptor interaction
NF-kappa B signaling pathway
TNF signaling pathway
Toll-like receptor signaling pathway
I
Pfirrmann grade II
Outgoing communication patterns of secreting cells
Incoming communication patterns of target cells
Cell groups
Patterns
Signaling

J

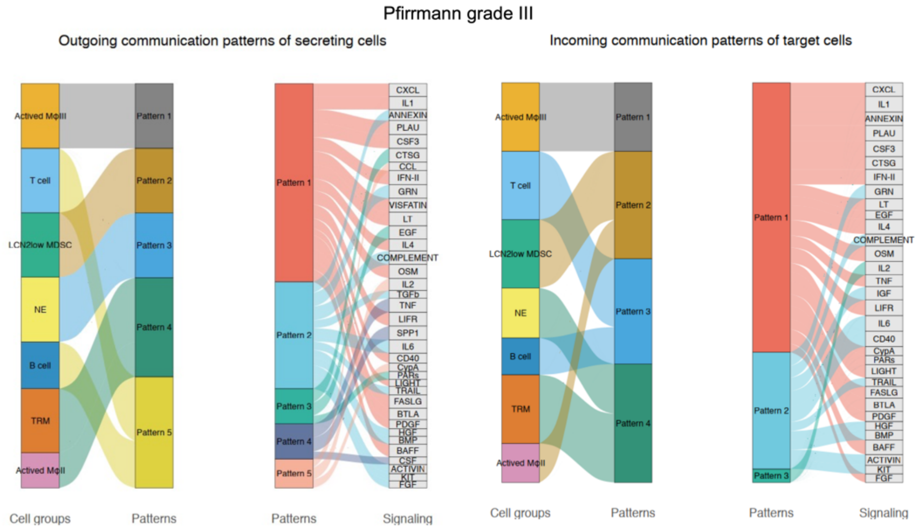

Figure S3. LCN2^{high} MDSCs mediate immune suppression during IVDD

(**A**) Heatmap presenting the expression levels of specific genes upon 8 cell subtypes: TRM, LCN2^{high} myeloid-derived suppressor cells (LCN2^{high} MDSCs), LCN2^{low} myeloid-derived suppressor cells (LCN2^{low} MDSCs), activated macrophage II (Activated Mφ II), activated macrophage III (Activated Mφ III), B cells, T cells, and neutrophils (NE). **(B)** UMAP visualization of reclustered activated Mφs and MDSCs from IVDD scRNA-seq data, resolving M1-like and M2-like subpopulations. **(C)** Stacked bar charts illustrating the ratio of each immune cell subpopulation across Pfirrmann grades I-V. **(D)** Split UMAP displaying the distribution of different immune cell subpopulations across Pfirrmann grades I-V, with LCN2^{high}MDSCs emergent and predominant at Pfirrmann II. **(E)** Box plot illustrating the cell differentiation potential calculated by Cytotrace, where higher values indicated greater differentiation capacity. **(F)** UMAP embedded with pseudotime values representing cell differentiation states. GSEAplot displayed GSEA enrichment analysis for LCN2^{high}MDSCs, with panel **(G)** showing the GO biological process enrichment analysis **(H)** presenting the KEGG pathway analysis. Sankey diagram illustrated the communication patterns of immune cell subpopulations, with (**I**) showing Pfirrmann grade II and (**J**) showing Pfirrmann grade III.

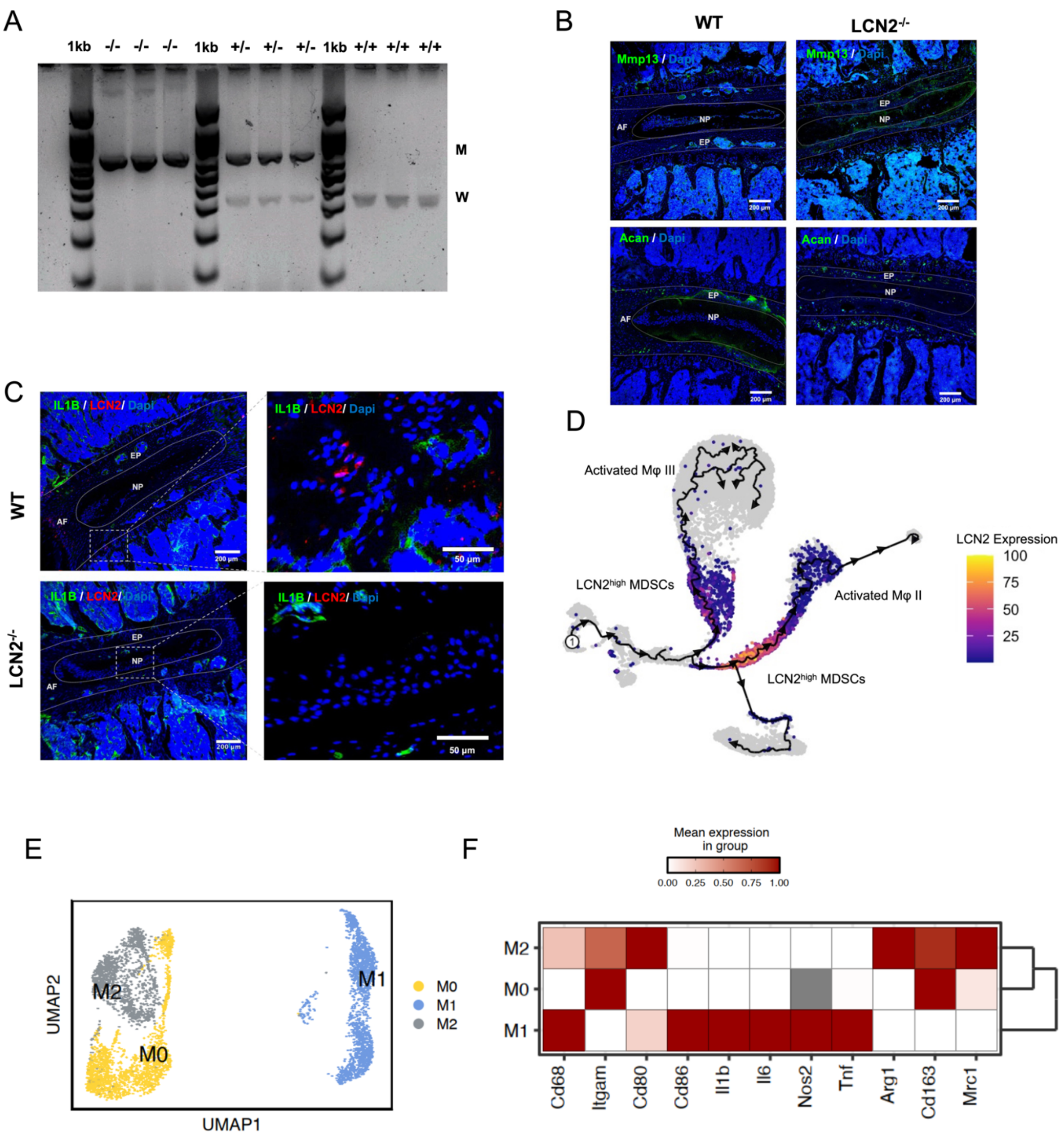

Figure S4. In vitro and in vivo assays investigating the effect of LCN2 on disc degeneration and macrophage polarization

(A) Genotyping results of LCN2-knockout (LCN2$^{-/-}$) mouse candidates. M: mutant allele, W: wild-type allele. **(B)** Immunofluorescence staining of aged (22-month-old) discs, showing elevated MMP13 and reduced Acan expression in LCN2$^{-/-}$ mice (scale bars=200 μm). **(C)** IL1B (green) and LCN2 (red) immunofluorescence in aged mice discs with disc margins outlined by dotted ovals (scale bars: left 200 μm, right 50 μm). **(D)** Monocle3-based pseudotime developmental trajectory revealed significant downregulation of LCN2 during the transition from LCN2^{high}MDSCs to Activated MφII clusters. **(E)** UMAP visualization of scRNA-seq data from M0/M1/M2 macrophages used to construct a reference panel for cell

type abundance analysis. **(F)** Heatmap showing expression levels of specific markers across macrophage subsets.
